# Adrenal fast-track and enhanced recovery in retroperitoneoscopic surgery for primary aldosteronism improving patient outcome and efficiency

**DOI:** 10.1007/s00345-024-04911-8

**Published:** 2024-03-22

**Authors:** Elle C. J. van de Wiel, Janneke Mulder, Anke Hendriks, Ingeborg Booij Liewes-Thelosen, Xiaoye Zhu, Hans Groenewoud, Peter F. A. Mulders, Jaap Deinum, Johan F. Langenhuijsen

**Affiliations:** 1 Department of Urology, Radboud University Medical Center, Nijmegen, The Netherlands; 2 Department of Anesthesiology, Radboud University Medical Center, Nijmegen, The Netherlands; 3 Department of Internal Medicine, Radboud University Medical Center, Nijmegen, The Netherlands; 4 Department for Health Evidence, Radboud University Medical Center, Nijmegen, The Netherlands

**Keywords:** Hyperaldosteronism, Adrenalectomy, Enhanced recovery after surgery, Perioperative care, Hospitalisation

## Abstract

**Purpose:**

No data exist on perioperative strategies for enhancing recovery after posterior retroperitoneoscopic adrenalectomy (PRA). Our objective was to determine whether a multimodality adrenal fast-track and enhanced recovery (AFTER) protocol for PRA can reduce recovery time, improve patient satisfaction and maintain safety.

**Methods:**

Thirty primary aldosteronism patients were included. Fifteen patients were treated with ‘standard-of-care’ PRA and compared with 15 in the AFTER protocol. The AFTER protocol contains: a preoperative information video, postoperative oral analgesics, early postoperative mobilisation and enteral feeding, and blood pressure monitoring at home. The primary outcome was recovery time. Secondary outcomes were length of hospital stay, postoperative pain and analgesics requirements, patient satisfaction, perioperative complications and quality of life (QoL).

**Results:**

Recovery time was much shorter in both groups than anticipated and was not significantly different (median 28 days). Postoperative length of hospital stay was significantly reduced in AFTER patients (mean 32 vs 42 h, CI 95%, *p* = 0.004). No significant differences were seen in pain, but less analgesics were used in the AFTER group. Satisfaction improved amongst AFTER patients for time of admission and postoperative visit to the outpatient clinic. There were no significant differences in complication rates or QoL.

**Conclusion:**

Despite no difference in recovery time between the two groups, probably due to small sample size, the AFTER protocol led to shorter hospital stays and less analgesic use after surgery, whilst maintaining and even enhancing patient satisfaction for several aspects of perioperative care. Complication rates and QoL are comparable to standard-of-care.

**Supplementary Information:**

The online version contains supplementary material available at 10.1007/s00345-024-04911-8.

## Introduction

Minimally invasive adrenalectomy is the standard of care for management of benign adrenal tumours (< 7 cm) [1, 2]. Two techniques are commonly practised either by transperitoneal or retroperitoneal access. Transperitoneal laparoscopic adrenalectomy (TLA) is usually performed with the patient in lateral decubitus position and has advantages of a familiar anatomy and wide surgical space [1]. Contrarily, posterior retroperitoneoscopic adrenalectomy (PRA) offers a more direct approach to the adrenal gland, avoiding intra-abdominal dissection and the need to move intra-abdominal organs. In a randomised controlled trial, Barczynski et al. showed several advantages of PRA over TLA, including shorter operating time, less blood loss and postoperative pain, faster recovery, improved cost-effectiveness and low risk of surgical access-site herniation [3]. We have previously shown significant differences in operating time and hospital stay in favour of PRA after a learning curve of approximately 70 procedures [4].

Despite these established differences between both techniques, patients undergoing PRA are preoperatively counselled and treated according to the same perioperative protocol as in TLA. However, we considered that PRA qualifies for so-called ‘fast-track surgery’ due to the estimated faster recovery compared with TLA [3, 4]. Fast-track surgery is an interdisciplinary, multimodality concept to accelerate postoperative recovery and has been safely introduced in various visceral and orthopaedic surgical procedures [5–8]. In the current literature, several advantages appear for fast-track surgery over standard surgical care in terms of early discharge from the hospital and short recovery time [9, 10].

Fast-track surgery is focussed on minimally invasive access to the operative field, optimised anaesthesia, targeted analgesics, stimulated early postoperative patient mobilisation, early postoperative oral feeding and avoidance of tubes and drains [11]. Also, preoperative patient education and counselling are important factors in prevention of postoperative complications and in reducing recovery time [12].

In this study, we have adjusted the perioperative protocol focussing on fast-track PRA. The aim is to evaluate the effect of modifying the perioperative care on recovery time, hospital stay, pain management and patients’ satisfaction after PRA.

## Materials and methods

### Patient selection

Patients with primary aldosteronism (PA) presenting at the outpatient urology clinic for adrenalectomy between September 2018 and November 2020, who met the inclusion criteria (Online Resource Table 1), were prospectively included in this observational study in which prespecified outcomes in the intervention group were compared with the ‘standard-of-care’ control group. This study design was chosen because a randomised design was not practically feasible. The first 15 patients included were treated according to the ‘standard-of-care’. The second 15 patients were treated according to the self-designed adrenal fast-track and enhanced recovery (AFTER) protocol. The research protocol was approved by the local Medical Ethics Committee.

### Perioperative protocol

Patients in the ‘standard-of-care’ group were counselled for surgery at the outpatient clinic at the discretion of the urologist without the use of a script. Patients in this group were standardly admitted on the day before surgery, they received non-standardised anaesthesia according to the anesthesiologists’ preference, an indwelling urinary catheter and postoperative patient-controlled analgesia (PCA) with morphine. Before discharge, patients were clinically assessed for the following discharge criteria, namely patients should be independent for ‘activities of daily living’ (ADL), sufficiently mobile and pain controlled with minimal oral analgesics. Two weeks after surgery, patients visited the nurse practitioner at the internal medicine outpatient clinic to evaluate blood pressure. Six weeks after surgery, patients had a surgical check at the urology outpatient clinic.

The AFTER protocol encompasses; a preoperative video (https://radboudumc.bbvms.com/view/default/3842248.html) that was created and distilled from interviews with a representative group of postsurgical PA patients of various ages and background. Further, this protocol includes admission on the day of surgery, standardised multimodality anaesthesia, a urinary catheter during surgery only, postoperative oral analgesics, early postoperative mobilisation and enteral feeding (stimulated by dedicated nurses at the ward). Further, early discharge was encouraged by instructing the patients to measure their blood pressure twice a day at home during 2 weeks after surgery. Blood pressure values were automatically transmitted to the electronic patient records with a communicating App (Whitings health mate). When diastolic or systolic blood pressure values were out of range, < 60 mmHg or > 110 mmHg for diastolic and < 100 mmHg or > 170 mmHg for systolic blood pressure, action was taken by the nurse practitioners, such as adjustment of medication. Two weeks postoperatively, patients visited the nurse practitioner at the internal medicine outpatient clinic. The nurse practitioner was trained to recognise complications and performed the postoperative surgical check including wound inspection and pain management during this consultation.

In both groups, adrenalectomy was performed retroperitoneoscopically. All operations were performed by an experienced urologist (JL, experienced in > 300 PRA procedures).

### Variables

Demographic and clinical variables included age, sex, body mass index (BMI), comorbidities, ASA status, tumour side and tumour diameter. The perioperative data included duration of surgery, anaesthesia technique and estimated blood loss. Post-operative in hospital data included length of stay (in hours), time until first oral intake (in hours), time until first mobilisation (in hours) and duration of the presence of a urinary catheter (in hours). The amount of pain was scored using the numerical rating scale (NRS) by a dedicated nurse. NRS-scores were taken until discharge at 4, 8, 12, 24, 36, 48 h after surgery. Analgesics consumed during hospital stay consisted of intravenous PCA morphine (only in the ‘standard-of-care’ group), and/or oral paracetamol, diclofenac and oxycodone.

We collected out of hospital data with questionnaires that were sent to every patient. Recovery was defined as the time point when patients experienced complete physical recovery and had fully resumed their normal daily activities. Furthermore, patients were asked for duration of analgesia requirement (sort of pain medication was not specified but only paracetamol and diclofenac were prescribed at discharge) and satisfaction with the perioperative protocol (measured by a self-designed rating scale of 0–100, see questionnaire in the supplementary material). The quality of Life (QoL) questionnaire, Short Form Health Survey (SF 36), was provided at 1 week before (baseline) and 6 weeks after surgery [13]. Complications < 30 days after surgery were registered according to the Clavien–Dindo classification [14].

### Statistical analyses

To compile comparable groups, we aimed for a balance of male/female ratio, mean BMI and ratio of left- and right-sided adrenalectomy between the intervention and control group. The control group was prospectively collected first and then sequentially the intervention group. As soon as addition of a patient to the latter group resulted in a significant imbalance, this patient was not included.

The primary outcome of this study was recovery time. Secondary outcomes were length of hospital stay, use of analgesics and pain scores after surgery, patient satisfaction, perioperative complications and QoL six weeks after surgery.

The prospective collected data were analysed with the use of SPSS 25.0 (Chicago IL, USA). We used Student *t* test to compare normally distributed continuous variables, the Mann–Whitney–Wilcoxon test for non-normally distributed variables and the chi‐square test for categorical variables. Statistical significance set at *P* < 0.05. The sample size was chosen based on a power of 80% to detect a difference between the two groups in recovery time of four days, which represents 10% of the recovery time that patients are usually counselled for (6 weeks = 42 days). Assuming an SD of four days, 15 patients were needed for enrolment in each group (two-sided α of 0.05). In case of dropout of patients, they were replaced with the same ratio in sex, BMI and side of adrenalectomy as in the inclusion group.

## Results

To balance the study groups, a female patient of the intervention group (inclusion number 13) was excluded and replaced by a male patient. Second, a patient in the intervention group with the indication for a right sided adrenalectomy (inclusion number 15) was excluded and replaced by a patient for left-sided adrenalectomy. Demographics and intra-operative variables were comparable between groups (Online Resource Table 2). One patient was replaced after dropping out from the AFTER group after conversion to transperitoneal adrenalectomy due to insufficient surgical progression and space restrictions.

In-hospital outcomes are shown in Table 1. There was a significant difference in total length of hospital stay (57 vs 32 h, *p* < 0.0001) and in postoperative length of hospital stay (42 vs 32, *p* = 0.004). In the AFTER group, 93.3% of the patients were discharged on the first day after surgery, compared to 53.3% in the control group. Inherent to the AFTER protocol duration of the urinary catheter, time until first mobilisation and first meal was significantly different between both groups. In the AFTER group, more patients received oral diclofenac and oxycodone on the day of surgery and on the first post-operative day compared to the control group which received PCA. Two perioperative complications were observed in each group. In the control group, one patient had a urinary tract infection for which antibiotic treatment was given and one patient experienced constipation for which an enema was given. In the AFTER group, two patients had a urinary tract infection for which antibiotic treatment was given. Post-operative pain scores are shown in Online Resource Fig. 1. There were no significant differences in pain scores between both groups on any postoperative time point.

**Table 1:** In-hospital outcomes

	Control group (*N* = 15)	AFTER group (*N* = 15)	*p*
**Duration of urinary catheter (h)**	22.8 ± 6.55	1.63 ± 0..23	PRD
**Time until first mobilisation (h)**	18.7 ± 7.01	4.0 ± 1.14	PRD
**Time until first meal (h)**	6.1 ± 4.07	3.2 ± 1.04	PRD
**Total length of hospital stay (h)**	56.5 ± 13.45	31.7 ± 8.04	< 0.0001*
**Postoperative length of hospital stay (h)**	42.1 ± 11.64	31.7 ± 8.04	0.004 *
**Discharge**			0.005**
Day 1 postoperative, *n* (%)	8 (53.3)	14 (93.3)	
Day 2 postoperative, *n* (%)	7 (47.7)	1 (6.7)	
**PCA analgesics first 24 h**, ***n*** **(%)**			N.A
No PCA	6 (40)	15 (100)	
PCA, 0 × used	1 (6.7)		
PCA, < 10 × used	4 (26.7)		
PCA, 10–20 × used	3 (20)		
PCA, > 20 × used	1 (6.7)		
**Analgesics use at day of surgery,** ***n*** **(%)**			N.A
PCM	12 (80)	4 (26.7)	
PCM + diclofenac	2 (13.3)	6 (40.0)	
PCM + diclofenac + oxycodone	0	4 (26.7)	
PCM + oxycodone	1 (6.7)	1 (6.7)	
**Analgesics use pod 1,** ***n*** **(%)**			N.A
None	1 (6.7)	0	
PCM	11 (73.3)	4 (26.7)	
PCM + diclofenac	2 (13.3)	9 (60.0)	
PCM + diclofenac + oxycodone	0	1 (6.7)	
PCM + oxycodone	1 (6.7)	1 (6.7)	
**Analgesics use pod 2**, ***n*** **(%)**			N.A
None	1	0	
PCM	4	0	
PCM + diclofenac	2	1	
PCM + diclofenac + oxycodone	0	0	
PCM + oxycodone	1	0	
**Complications**			1.000**
None	13	13	
Clavien–Dindo I	0	0	
Clavien–Dindo II	2	2	

*h* hours, *N.A.* not applicable, *PCA* Patient-Controlled Analgesia, *PCM* paracetamol, pod post-operative day, *PRD* protocol-related differences

Categorical variables are presented as *n* (%); continuous variables are presented as mean ± SD

*Group differences were tested with the independent samples *t*-test

**Group differences were tested with the Mann–Whitney *U* test

In the AFTER group, one patient required medication adjustment due to excessive systolic blood pressure measured on the first day at home. Recovery outcomes are shown in Table 2. Patients in both groups recovered after a median of 28 days. There was no significant difference between both groups in the number of days patients used analgesics at home and the number of days until they were pain-free.

**Table 2:** Recovery outcomes

	Control group (*N* = 15)	AFTER group (*N* = 15)	P*
Recovery (days)	28 [8–42]	28 [2–49]	0.713
Analgesics used at home (days)	5 [0–28]	4 [0–10]	0.489
Time until pain-free (days)	14 [0–42]	10 [0–30]	0.161

Variables are denoted as median [range]

*Group differences were tested with the Mann–Whitney *U* test

Patient satisfaction scores are shown in Online Resource Table 3. Patients in the AFTER group were more satisfied about the preoperative admission. Both groups experienced the indwelling urinary catheter as neutral despite the differences in duration of the catheter staying in place. Patients in both groups were equally satisfied about the length of hospital stay. Patients in the AFTER group were more satisfied with their post-operative visit to the internal medicine outpatient clinic only compared with the patients in the control group with an extra post-operative visit after 6 weeks to the urology outpatient clinic. The extra visit to the urology outpatient clinic was not missed by most patients (80%) in the AFTER group. Finally, patients in both groups were equally satisfied about the whole surgical path, which includes all outpatient clinic visits, day of surgery, hospital stay and the postoperative care.

Online Resource Table 4 shows that postoperative HRQoL scores of some subscales were significantly higher in the respective groups (bold subscales). There were no significant differences in the delta of the subscales.

## Discussion

In this study, we modified the perioperative care for PRA in PA patients into a fast-track protocol. Although the primary outcome, recovery time, did not differ, the AFTER protocol contributes to a reduction in the length of hospital stay whilst maintaining adequate pain management with high or even improved patient satisfaction. Complication rates and QoL s6 weeks after surgery are comparable to that in the ‘standard-of-care’ group.

Although the primary endpoint of a difference in recovery time is not achieved, the fast-track protocol does provide more efficient perioperative care with shorter admission time and fewer outpatient visits. Measurement of recovery time is complex and involves multiple domains, such as physical recovery, mental recovery and recovery from pain, whilst in literature, this is often bypassed by choosing return to work as endpoint [15, 16]. In both study groups, recovery time was 28 days. This may seem long after retroperitoneal surgery but may be explained by our choice of full recovery time as primary endpoint. Based on experience in transperitoneal adrenalectomy, we assumed recovery time to be 42 days in the power calculation. The lower recovery time of 28 days probably explains the non-significant outcome in primary endpoint because the probability of a substantial difference may then decrease.

The same surgical approach was performed in both study groups resulting in low pain scores on each postoperative time point, which were not significantly different. However, the AFTER patients did not receive intravenous morphine and therefore it may be assumed that PCA can be abandoned after PRA. We show that stimulation of early feeding and mobilisation after retroperitoneoscopic surgery are feasible and have been shown to be important components to minimise complications after surgery [17, 18].

We conclude that the short hospital stay in the AFTER group is due to multimodality changes compared to the ‘standard-of-care’ group. First, the improved preoperative patient information with an informative video plays an important role in modifying the patient’s expectation of postoperative recovery. In other studies, it was demonstrated that preoperative education (verbal, written or audio–visual) will aid to reduce preoperative anxiety and postoperative pain and may also enhance postsurgical recovery [19, 20]. Second, the early removal of the urinary catheter, together with the early mobilisation and enteral feeding, resulted in instant ambulation. Combined with home monitoring of blood pressures, these measures facilitated the patients’ early discharge.

The satisfaction questionnaires show some clinically relevant findings. Patients in the AFTER group were more satisfied with the same day admission for surgery, which now seems to be justified based on our results. Furthermore, both groups experienced comparable bothersome feelings by the urinary catheter and the complications of urinary tract infections despite the removal of the catheter directly after surgery in the AFTER group. Therefore, we consider it justified not to routinely insert urinary catheters any more in PRA patients. Patients in the AFTER group were more satisfied with the reduced postoperative check due to the combined outpatient clinic visit for surgical care with the familiar nurse practitioner of internal medicine saving an extra visit to the hospital.

Although a formal cost evaluation was not included in the study, it is likely that the AFTER protocol reduces costs since patients were hospitalised one day less and included only one outpatient clinic check. The cost reduction in our setting is 800 euro per patient.

There are several strengths of our study such as the prospective nature with accurate data collection. Further, the preoperative information video, which was designed in cooperation with PA patients, is a new modality for counselling PRA patients. Video is a powerful tool for informing patients in an understandable way compared to an information leaflet that lacks the audio–visual aspects. Limitations of this study are the small sample size from a single high-volume institution and a single albeit highly experienced adrenal surgeon which could make our results difficult to extrapolate to other centres. Therefore, further validation of the protocol in other hospitals, for instance with a cluster-randomised design, is warranted. Finally, the unavoidable non-randomised nature of this study has a risk of selection bias and reduces the external validity of the study. But since the study groups are well weighted, it seems that the risk of bias is limited.

Because of the positive results and high satisfaction, we have now adopted the AFTER protocol as standard of care in our hospital. Following validation, the protocol can be applied in other centres performing PRA. The next question this study raises is whether we could offer PRA as a day care procedure. With appropriate patient selection, day care adrenalectomy has the potential to decrease costs, improve inpatients capacity and decrease patients’ exposure to hospital-acquired conditions [21, 22].

## Conclusions

In conclusion, although recovery time does not differ, the AFTER protocol increases efficiency after PRA in PA patients with reduced hospital stay and less requirement of analgesics after surgery, whilst maintaining high or even improved patient satisfaction for several aspects of perioperative care. Complication rates and QoL are comparable to the standard-of-care.

## Supplementary Information

Below is the link to the electronic supplementary material.

Supplementary file1 (DOCX 132 KB)

## Data Availability

The data from this study is available upon request.

## References

[1] Raffaelli M, De Crea C, Bellantone R (2019) Laparoscopic adrenalectomy. Gland Surg 8(Suppl 1):S41-s52 31404184 10.21037/gs.2019.06.07 PMC6646817

[2] Assalia A, Gagner M (2004) Laparoscopic adrenalectomy. Br J Surg 91(10):1259–1274 15376201 10.1002/bjs.4738

[3] Barczynski M, Konturek A, Nowak W (2014) Randomized clinical trial of posterior retroperitoneoscopic adrenalectomy versus lateral transperitoneal laparoscopic adrenalectomy with a 5-year follow-up. Ann Surg 260(5):740–747 (**discussion 747-8**) 25243546 10.1097/SLA.0000000000000982

[4] van Uitert A et al (2017) Evaluating the learning curve for retroperitoneoscopic adrenalectomy in a high-volume center for laparoscopic adrenal surgery. Surg Endosc 31(7):2771–2775 27752814 10.1007/s00464-016-5284-0 PMC5487835

[5] Kehlet H, Wilmore DW (2008) Evidence-based surgical care and the evolution of fast-track surgery. Ann Surg 248(2):189–198 18650627 10.1097/SLA.0b013e31817f2c1a

[6] Raphael M, Jaeger M, van Vlymen J (2011) Easily adoptable total joint arthroplasty program allows discharge home in two days. Can J Anaesth 58(10):902–910 21822756 10.1007/s12630-011-9565-8

[7] Melnyk M et al (2011) Enhanced recovery after surgery (ERAS) protocols: Time to change practice? Can Urol Assoc J 5(5):342–348 22031616 10.5489/cuaj.11002 PMC3202008

[8] Collins JW et al (2016) Enhanced recovery after robot-assisted radical cystectomy: EAU Robotic Urology Section Scientific Working Group Consensus View. Eur Urol 70(4):649–660 27234997 10.1016/j.eururo.2016.05.020

[9] Parisi A et al (2020) Enhanced recovery after surgery (ERAS): a systematic review of randomised controlled trials (RCTs) in bariatric surgery. Obes Surg 30(12):5071–5085 32981000 10.1007/s11695-020-05000-6

[10] Zhu S et al (2017) Enhanced recovery after surgery for hip and knee arthroplasty: a systematic review and meta-analysis. Postgrad Med J 93(1106):736–742 28751437 10.1136/postgradmedj-2017-134991 PMC5740550

[11] Kehlet H, Wilmore DW (2002) Multimodal strategies to improve surgical outcome. Am J Surg 183(6):630–641 12095591 10.1016/s0002-9610(02)00866-8

[12] Klaiber U et al (2018) Impact of preoperative patient education on the prevention of postoperative complications after major visceral surgery: the cluster randomized controlled PEDUCAT trial. Trials 19(1):288 29793527 10.1186/s13063-018-2676-6 PMC5968532

[13] VanderZee KI et al (1996) Psychometric qualities of the RAND 36-Item Health Survey 1.0: a multidimensional measure of general health status. Int J Behav Med 3(2):104–122 16250758 10.1207/s15327558ijbm0302_2

[14] Dindo D, Demartines N, Clavien PA (2004) Classification of surgical complications: a new proposal with evaluation in a cohort of 6336 patients and results of a survey. Ann Surg 240(2):205–213 15273542 10.1097/01.sla.0000133083.54934.ae PMC1360123

[15] Larson DB et al (2017) Return to normal activities and work after living donor laparoscopic nephrectomy. Clin Transplant. 10.1111/ctr.12862 27740731 10.1111/ctr.12862

[16] Sultan R et al (2006) Time to return to work and physical activity following open radical retropubic prostatectomy. J Urol 176(4 Pt 1):1420–1423 16952648 10.1016/j.juro.2006.06.011

[17] Hu Y, McArthur A, Yu Z (2019) Early postoperative mobilization in patients undergoing abdominal surgery: a best practice implementation project. JBI Evid Synth 17(12):2591–2611 10.11124/JBISRIR-D-19-00063 31725070

[18] Varadhan KK, Lobo DN, Ljungqvist O (2010) Enhanced recovery after surgery: the future of improving surgical care. Crit Care Clin 26(3):527–547 (**x**) 20643305 10.1016/j.ccc.2010.04.003

[19] Daltroy LH et al (1998) Preoperative education for total hip and knee replacement patients. Arthritis Rheum 11(6):469–478 10.1002/art.1790110607 10030179

[20] Egbert LD et al (1964) Reduction of postoperative pain by encouragement and instruction of patients. N Engl J Med 270(16):825–827 14108087 10.1056/NEJM196404162701606

[21] Pigg RA et al (2022) Patient satisfaction is equivalent for inpatient and outpatient minimally-invasive adrenalectomy. J Surg Res 269:207–211 34601371 10.1016/j.jss.2021.08.019 PMC8688216

[22] Mohammad WM, Frost I, Moonje V (2009) Outpatient laparoscopic adrenalectomy: a Canadian experience. Surg Laparosc Endosc Percutan Tech 19(4):336–337 19692886 10.1097/SLE.0b013e3181b05dcc

