# Supplementary material for: Adrenal fast-track and enhanced recovery in retroperitoneoscopic surgery for primary aldosteronism improving patient outcome and efficiency
Source: World J Urol. 2024 Mar 22;42(1):187. doi: 10.1007/s00345-024-04911-8 (PMC10959772; doi:10.1007/s00345-024-04911-8)
Supplement: Supplementary file 1 — Supplementary file1 (DOCX 132 KB) [file 345_2024_4911_MOESM1_ESM.docx]

DATA supplement

This appendix has been provided by the authors to give readers additional information about their work.

Supplement to:

**Adrenal fast-track and enhanced recovery in retroperitoneoscopic surgery for primary aldosteronism improving patient outcome and efficiency**

Journal: world journal of urology

Authors:

Elle van de Wiel^1^ MD, Janneke Mulder^2^ MD, Anke Hendriks^3^ , Ingeborg Booij Liewes-Thelosen^3^, Xiaoye Zhu^1^ MD PhD, Hans Groenewoud^4^ MSc, Professor Peter Mulders^1^ MD, Jaap Deinum^3^ MD PhD, Johan Langenhuijsen^1^ MD PhD

1. Radboud University Medical Center, Department of Urology, Nijmegen, the Netherlands
2. Radboud University Medical Center, Department of Anesthesiology, Nijmegen, the Netherlands
3. Radboud University Medical Center, Department of Internal Medicine, Nijmegen, the Netherlands
4. Radboud University Medical Center, Department for Health Evidence, Nijmegen, the Netherlands

**Corresponding author:** E.C.J. van de Wiel, Radboud University Medical Center, Department of Urology, P.O. box 9101, 6500 HB Nijmegen, The Netherlands, telephone: +31 (0)24 361 9515, fax +31 (0)24 3635121, e-mail: [elle.vandewiel@radboudumc.nl](mailto:elle.vandewiel@radboudumc.nl)

**Table 1 Patient eligibility criteria**

| Patient Criteria |
| --- |
| Primary aldosteronism |
| Age > 18 |
| BMI <35 |
| ASA ≤ III |
| Possess and be able to work with a smartphone |
| Dutch speaking |
| Able to provide informed consent |

**Results**

**Table 2: Demographics**

|  | **Control group (N=15)** | **AFTER group (N=15)** | **P*** |
| --- | --- | --- | --- |
| Gender **  Male  Female | 5 (33.3)  10 (66.7) | 7 (46.7)  8 (53.3) | 0.456 |
| Age during surgery (years) * | 51,80 ± 11,2 | 51,93 ± 6,5 | 0.181 |
| ASA **  I, II  III | 12 (80)  3 (20) | 12 (80)  3 (20) | 1.000 |
| BMI (kg/m^2^) * | 27,47 ± 4,80 | 27,53 ± 3.00 | 0.124 |
| Comorbidities **  None  Cardiac disease  OSAS  Diabetes  Neurologic  Other | 9 (60)  0  2 (13.3)  1 (6.7)  2 (13.3)  1 (6.7) | 11 (73.3)  1 (6.7)  2 (13.3)  0  1 (6.7)  0 | 0.618 |
| Side **  Right  Left | 11 (64.7%)  4 (30.8%) | 6 (35.3%)  9 (69.2%) | 0.065 |
| Diameter tumor (cm) * | 1,54 ± 0.69 | 1,52 ± 0.82 | 0.392 |
| Intraoperative *  Blood loss (ml)  Duration of surgery (min) | 5,33 ± 1,3  52,33 ± 19,6 | 5,33 ± 1,3  48,07 ± 16,1 | 1.000  0.494 |

*Categorical variables are presented as n (%); continuous variables are presented as mean ± SD. *Group differences were tested with the independent samples t-test. **Group differences were tested with the Mann-Whitney U test. ASA = American Society of Anesthesiologists, BMI = body mass index, OSAS = Obstructive Sleep Apnea Syndrome.*

**Table 3: Satisfaction scores**

|  | Control group (N=15) | AFTER group (N=15) | P* |
| --- | --- | --- | --- |
| Preoperative outpatient visit to urologist | 93,33 ± 11,4 | 91,67 ± 15,43 | 0,367 |
| Preoperative admission | 82,00 ± 27,44 | 98,33 ± 6,46 | <0.0001 |
| Indwelling urinary catheter duration | 48,33 ± 35,94 | 48,33 ± 32,00 | 0.427 |
| Length of hospital stay | 53,33 ± 8,98 | 56,67 ± 17,59 | 0.132 |
| Analgesics during hospital stay | 88,67 ± 20,66 | 81,67 ± 30,57 | 0.153 |
| Postoperative check  Urology  Internal medicine | 80 ± 23,53 | 93,33 ± 14,84 | 0.003 |
| Did you miss a postoperative check by the urologist?  Yes  No |  | 3 (20%)  12 (80%) |  |
| Satisfaction whole surgical path | 87,67 ± 20,34 | 83,33 ± 26,16 | 0.343 |

*Scores: 0-20 =* very unsatisfied, 20-40 = unsatisfied, 40-60 = neither satisfied nor unsatisfied, 60-80 = satisfied, 80-100 = very satisfied

*Continuous variables are presented as mean ± SD****.*** **Group differences were tested with the independent samples t-test.*

**Table 4: SF 36 – HRQoL**

| SF-36 subscales control group | Baseline | Post-operative | P* (paired t-test) | Delta |
| --- | --- | --- | --- | --- |
| **Physical functioning** | **71,33 ± 20,82** | **84,33 ± 19,44** | **0.002** | 13 ± 14,98 |
| Role limitations due to physical health (RP) | 33,33 ± 43,98 | 49,17 ± 44,94 | 0.167 | 15,83 ± 49,66 |
| Role limitations due to emotional health | 53,33 ± 43,28 | 84,44 ± 35,34 | 0.825 | 31,11 ± 54,14 |
| Vitality | 35,00 ± 24,35 | 62,33 ± 18,31 | 0.104 | 27,33 ± 23,21 |
| **Mental health (emotional wellbeing)** | **62,40 ± 23,36** | **75,73 ± 17,20** | **0.018** | 13,33 ± 18,92 |
| Social functioning | 56,67 ± 28,69 | 74,17 ± 22,87 | 0.122 | 17,5 ± 28,27 |
| Bodily pain | 79,67 ± 22,99 | 79,83 ± 21,68 | 0.107 | 0.17 ± 23,80 |
| **General health** | **50,67 ± 18,31** | **65,00 ± 20,09** | **0.036** | 14.33 ± 18,41 |

| SF-36 subscales AFTER group | Baseline | Post-operative | P* | Delta | P** delta |
| --- | --- | --- | --- | --- | --- |
| **Physical functioning** | **78,33 ± 19,51** | **89,33 ± 14,74** | **<0.0001** | 11 ± 11,83 | 0.688 |
| Role limitations due to physical health (RP) | 58,33 ± 46,92 | 63,33 ± 44,19 | 0.381 | 5 ± 56,06 | 0.580 |
| Role limitations due to emotional health | 77,77 ± 34,88 | 91,11 ± 26,63 | 0.840 | 13,33 ± 45,07 | 0.337 |
| **Vitality** | **51,00 ± 27,27** | **66,33 ± 19,41** | **0.016** | 15,33 ± 21,83 | 0.156 |
| **Mental health (emotional wellbeing)** | **70,67 ± 22,86** | **81,07 ± 17,14** | **0.009** | 10,4 ± 17,49 | 0.663 |
| Social functioning | 75,00 ± 32,73 | 89,17 ± 15,57 | 0.532 | 14,17 ± 38,63 | 0.790 |
| Bodily pain | 84,83 ± 19,19 | 87,00 ± 20,03 | 0.615 | 2,17 ± 25,70 | 0.827 |
| **General health** | **61,33 ± 19,32** | **74,33 ± 28,89** | **0.031** | 13 ± 18 | 0.843 |

*Continuous variables are presented as mean ± SD. *Group differences were tested with the paired samples t-test. **Group differences were tested with the independent samples t-test.*


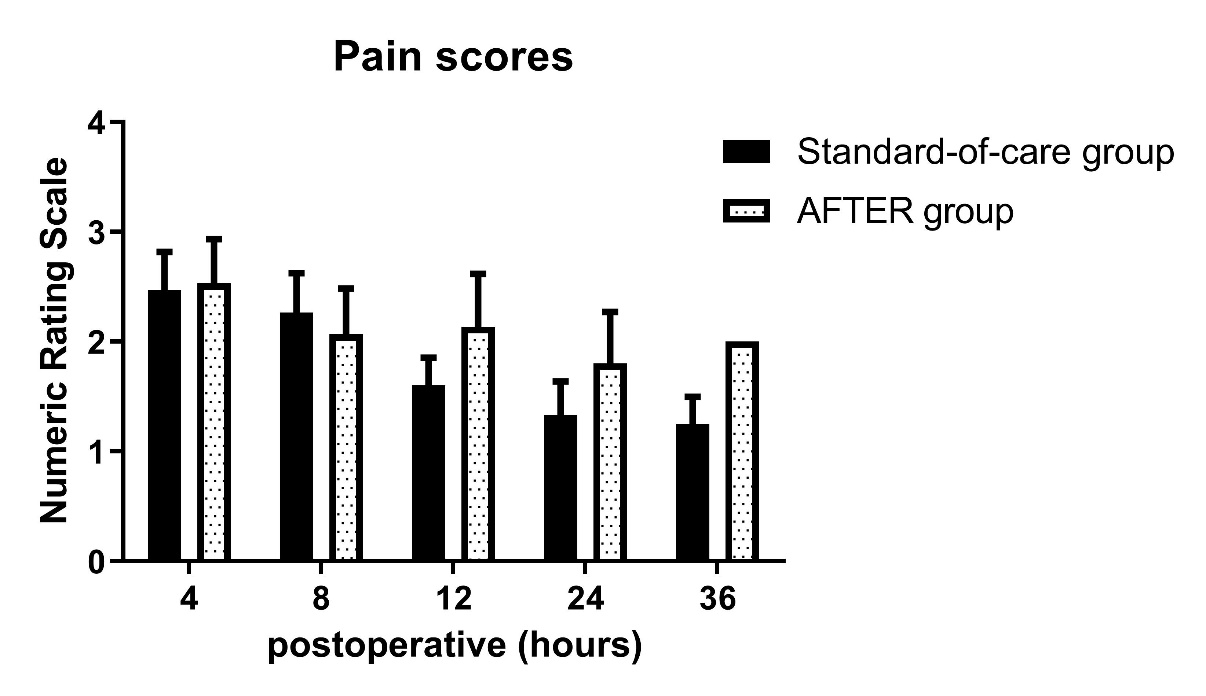


**Figure 1: postoperative pain scores**

**Satisfaction questionnaire**

The questions below are designed to measure your satisfaction with the peri operative adrenal surgery protocol. For each question, you should circle the number that best describes your satisfaction level.

1. **Outpatient visit urologist pre-operatively.** Were you satisfied with the outpatient clinic visit? This includes the appointment with the urologist before the surgery in which the surgery was explained.

| 1 | 2 | 3 | 4 | 5 |
| --- | --- | --- | --- | --- |

very unsatisfied unsatisfied neither satisfied satisfied very satisfied

nor unsatisfied

1. **Admission before surgery.** You were admitted "1 day before surgery" / "the day of surgery" (delete as appropriate). Were you satisfied with the length of admission before surgery?

| 1 | 2 | 3 | 4 | 5 |
| --- | --- | --- | --- | --- |

very unsatisfied unsatisfied neither satisfied satisfied very satisfied

nor unsatisfied

1. **Urinary catheter during admission.** You had a urinary catheter during admission: “yes”/”no” (delete as appropriate).

**a** Fill in if you have had a catheter: How did you experience the catheter? If you have experienced the catheter as very annoying, choose number 1. If you have experienced the catheter not annoying at all, select number 5**.**

| 1 | 2 | 3 | 4 | 5 |
| --- | --- | --- | --- | --- |

very annoying neutral not annoying at all

b Fill in if you did not have a catheter: How did you experienced not having a catheter after surgery?

| 1 | 2 | 3 | 4 | 5 |
| --- | --- | --- | --- | --- |

very annoying neutral not annoying at all

1. **Length of hospital stay**. A score of 1 means you thought the length of hospital stay was too long. A score of 5 means that you thought the length of hospital stay was too short. A score of 3 means that you were satisfied with the length of hospital stay.

| 1 | 2 | 3 | 4 | 5 |
| --- | --- | --- | --- | --- |

too long satisfied too short

1. **Pain relief mediation.** Were you satisfied with the pain relief during admission?

| 1 | 2 | 3 | 4 | 5 |
| --- | --- | --- | --- | --- |

very unsatisfied unsatisfied neither satisfied satisfied very satisfied

nor unsatisfied

1. **Outpatient visit urologist post-operatively (Fill in 6a & 6b if you have visited the urologist after the operation).**
   1. How useful did you find the outpatient visit with the urologist 6 weeks after surgery?

| 1 | 2 | 3 | 4 | 5 |
| --- | --- | --- | --- | --- |

Not useful at all neutral very useful

b. Suppose that the urologist's outpatient visit, 6 weeks after surgery, was replaced by a check-up by an internal medicine specialized nurse. Would you be satisfied with this?

| 1 | 2 | 3 | 4 | 5 |
| --- | --- | --- | --- | --- |

very unsatisfied unsatisfied neither satisfied satisfied very satisfied

nor unsatisfied

1. **Outpatient visit internal medicine, specialized nurse (Fill in 7a & 7b if you have not visited the urologist after the operation).**

a. Were you satisfied with the check-up by the specialized nurse 2 weeks after the operation? (This includes the check where the wound was also checked).

| 1 | 2 | 3 | 4 | 5 |
| --- | --- | --- | --- | --- |

very unsatisfied unsatisfied neither satisfied satisfied very satisfied

nor unsatisfied

b. Did you miss a visit at the urologist a few weeks after the operation?

- Yes
- No

1. **How satisfied are you with the total surgery path?** This includes all hospital visits, surgery, hospital stay and postoperative care**.**

| 1 | 2 | 3 | 4 | 5 |
| --- | --- | --- | --- | --- |

very unsatisfied unsatisfied neither satisfied satisfied very satisfied

nor unsatisfied
